# The Impact of Trimetazidine on Disease Severity and Quality of Life in Parkinson’s Disease

**DOI:** 10.1038/s41598-020-66692-5

**Published:** 2020-06-22

**Authors:** Dávid Pintér, Annamária Juhász, Márk Harmat, József Janszky, Norbert Kovács

**Affiliations:** 1 Department of Neurology, University of Pécs, Medical School, Pécs, Hungary; 2 MTA-PTE Clinical Neuroscience MR Research Group, Pécs, Hungary

**Keywords:** Neurology, Neurological disorders, Neurodegenerative diseases, Parkinson's disease

## Abstract

Trimetazidine is contraindicated in movement disorders, however, a not negligible part of trimetazidine users is still patients with Parkinson’s disease (PD). The present study aimed to objectively determine the impact of trimetazidine on the severity of symptoms and the health-related quality of life of patients with PD by measuring changes after its withdrawal. A consecutive series of 42 patients with PD using trimetazidine underwent detailed neurological and neuropsychological assessments at baseline and three months after the discontinuation of trimetazidine. Clinically relevant improvements were achieved with discontinuation of trimetazidine according to changes in scores of each part of the Movement Disorder Society-sponsored Unified Parkinson’s Disease Rating Scale (Part I: −25.7%, p < 0.001; Part II: −23.8%, p < 0.001; Part III: −28.5%, p < 0.001; Part IV: −30.1%, p = 0.004) and total scores of the Non-Motor Symptoms Scale (−25.6%, p = 0.004) and the Montgomery-Asberg-Depression Rating Scale (−20.1%, p = 0.001). Benefits resulting from the withdrawal of the drug also manifested in the improvement of the health-related quality of life based on changes in the summary index of the 39-item Parkinson’s Disease Questionnaire (−18.2%, p = 0.031). Our results provide clinical rationale for strictly avoiding the use of trimetazidine in PD. Discontinuation of trimetazidin results in clinically relevant improvements in Parkinsonian symptoms.

## Introduction

Antipsychotics are the best known causes of drug-induced parkinsonism and medication-related symptom deterioration of patients with Parkinson’s disease (PD)^1^. However, it is a frequently forgotten fact that a number of other commonly used medications, such as antiemetics (e.g., metoclopramide), drugs for preventive migraine therapy (e.g., flunarizine, and cinnarizine), antiepileptic (e.g., valproate) and antianginal drugs (e.g., trimetazidine), may also be responsible for drug-induced movement disorders and worsening of Parkinsonian symptoms^2^.

Trimetazidine (1-[2,3,4-trimethoxibenzyl]-piperazine, TMZ) is a widely used add-on therapy for the symptomatic treatment of stable coronary heart disease^3–5^. In addition to some rare side effects associated with TMZ treatment, including nausea, vomiting, epigastric pain, headache, liver dysfunction, thrombocytopenia, and agranulocytosis^6,7^; ample evidence exists for adverse effects of the drug on motor function. TMZ can induce reversible parkinsonism, tremor, and orofacial dyskinesia^5,7–13^; and aggravate symptoms of existing movement disorders including PD^5,7^. These effects of the drug might result from its piperazine core^10^, which can also be found in prochlorperazine and fluphenazine, antipsychotics reported to induce parkinsonism and worsen PD^1,14^. Drugs owning a piperazine core can process strong postsynaptic inhibition of dopamine receptors, including dopamine D2 receptors^10^, which are present in many brain structures such as the striatum having a substantial role in regulation of movements. The potential antipsychotic-like effect of TMZ described in a rodent model may also support the hypothesis that TMZ can cross the blood-brain barrier and interact with dopamine receptors in the central nervous system^15^.

Based on available findings, the European Medicines Agency (EMA) made a recommendation for the more careful use of TMZ in certain groups of patients and against the application of the drug for the treatment of patients with PD in 2012^16^. However, some recent data show that this recommendation is not followed strictly enough since a not negligible part of TMZ users is still patients with movement disorders diagnosed prior to the prescription of the drug^13^.

Although motor symptoms of reversible TMZ-induced parkinsonism have been considered to be mild by previous descriptions^5,7,9,11^, they can have a serious impact on the health-related quality of life (HRQoL)^5^. However, parkinsonism induced or aggravated by TMZ treatment is also characterized by a sustained complete symptomatic remission or improvement after the discontinuation of the drug;^5,8,9,11^ therefore, withdrawal of TMZ may theoretically improve the HRQoL of patients both with reversible parkinsonism and PD aggravated by the drug.

This single-center, prospective, longitudinal study aimed to objectively evaluate the impact of TMZ treatment on the severity of Parkinsonian symptoms and the HRQoL of subjects with PD. We tried to explore the effects of discontinuation of TMZ.

## Materials and Methods

### Study population and protocol

All study-related procedures were approved by the Regional and Institutional Ethical Committee (3617.316-24987/KK41) and performed in accordance with the Declaration of Helsinki.

The present study included a consecutive series of 42 patients with the clinical diagnosis of PD based on the UK Brain Bank criteria^17,18^ and concomitant TMZ treatment who presented at the Department of Neurology, University of Pécs, Hungary between 2014 and 2018. TMZ treatment (initiation of the drug in subjects with previously established PD or ongoing TMZ treatment after the diagnosis of PD) had been applied independently of the authors. The University of Pécs is a tertiary movement disorders center, and many of the participants were referred for further treatment. Each patient was examined immediately after learning the concomitant use of TMZ (baseline examination, BL).

After obtaining an individual written informed consent for participating in the study, all enrolled patients underwent detailed neurological and neuropsychological assessments at BL. None of the enrolled subjects was treated with other medications (e.g., antipsychotics, calcium channel blockers, and antiemetics) which could have worsened the symptoms of PD. Subsequently, TMZ treatment was discontinued, and patients were reevaluated 3 months after the withdrawal of the drug (follow-up, FU) based on the recommendation given by the EMA^16^. Oral antiparkinsonian treatment remained stable in all subjects in the interval between the BL and 3-month FU visits. The included patients were followed for at least a 12-month period after the withdrawal of TMZ.

### Assessed scales

In addition to demographic data (e.g., age, sex, and level of education), disease-specific data (e.g., duration of disease defined as the interval between the first observed PD-related motor problems and the time of BL examinations) and type of PD (tremor dominant, rigid-akinetic or mixed type), the severity of disease was globally measured by the Hungarian validated version of the Movement Disorders Society-sponsored Unified Parkinson’s Disease Rating Scale (MDS-UPDRS)^19,20^. Based on Part III of the MDS-UPDRS, an axial score (postural instability and gait difficulty, PIGD) was calculated^21^. The nested version of the Hoehn-Yahr Scale (HYS), which is included in the MDS-UPDRS, was also obtained; and the patients were categorized based on the overall disease severity as mild (HYS 1&2), moderate (HYS 3) and severe (HYS 4&5)^22^.

For the better characterization of nonmotor symptoms of the study population, the Hungarian validated versions of the Non-Motor Symptoms Scale (NMSS)^23^, the Montgomery-Asberg Depression Rating Scale (MADRS)^24^, the Parkinson’s Disease Sleep Scale 2^nd^ version^25^, and the Epworth Sleepiness Scale^26^ were used. Cognitive performance was measured by the Hungarian validated versions (7.1, 7.2 and 7.3) of the Montreal Cognitive Assessment^27^.

To measure the HRQoL, the Hungarian validated version of the 39-item Parkinson’s Disease Questionnaire (PDQ-39)^28^ was applied.

All the scales were assessed both during the BL and the FU visits.

### Statistical analysis

To test normality, the Shapiro-Wilk test was used. Because most of the data from the applied scales followed the normal distribution, mean and standard deviation values were calculated. For the analysis of changes in scores of scales due to TMZ withdrawal, the paired samples t-test and the Wilcoxon signed rank test were utilized. For categorical variables (e.g., HYS), the Chi-square test was applied. The level of statistical significance was set at 0.05. All statistical analyses were performed using the IBM SPSS software package (version 24.0.2, IBM Inc., Armonk, NY, USA).

## Results

Sociodemographic and disease-specific data of the study population at BL are demonstrated in Table 1.

**Table 1:** Baseline characteristics for the study cohort (n = 42).

Characteristic		Mean ± standard deviation or n (%)
Age (years)		71.1 ± 8.1
Males		23 (54.8)
Right handed subjects		41 (97.6)
Education (years)		11.0 ± 3.0
Duration of disease (years)		3.3 ± 3.8
Duration of levodopa use (years)		3.0 ± 4.3
Levodopa LED (mg)		291.7 ± 468.2
Dopamine agonist LED (mg)		99.0 ± 227.1
Total LED (mg)		411.0 ± 582.4
Severity of disease	Mild (HYS 1&2)	25 (59.5)
	Moderate (HYS 3)	9 (21.4)
	Severe (HYS 4&5)	8 (19.1)

Abbreviations: HYS = Hoehn-Yahr Scale; LED = levodopa equivalent dosage.

The mean duration of TMZ use, defined as the interval between initiation of TMZ and the BL visit, was 78.5 ± 24.9 months. The mean daily administered dose of the drug at BL was 72.1 ± 9.8 mg.

Changes in the severity of motor and nonmotor symptoms of PD and the level of the HRQoL due to discontinuation of TMZ are presented in Table 2. There were significant decreases of 4.0, 3.5, 10.4 and 1.2 points in Parts I, II, III and IV of the MDS-UPDRS, respectively. Change in the PIGD score (3.1 points) indicated a notable improvement in the severity of axial symptoms, including disturbances of posture, gait problems, and postural instability. Among others, the improvement of axial symptoms led to a significant reduction in HYS. None of the patients suffered from reversible parkinsonism, however, antiparkinsonian treatment led to complete disappearance of motor symptoms (HYS 0) in two patients after the discontinuation of TMZ. In these two cases, discontinuation of antiparkinsonian medications resulted in the reemergence of Parkinsonian symptoms.

**Table 2:** Changes in scores of the applied scales due to discontinuation of trimetazidine.

		Baseline [mean ± SD or n (%)]	Follow-up^a^ [mean ± SD or n (%)]	Change		p-value
				Absolute (mean ± SD)	Relative change (%)^b^ (mean)	
MDS-UPDRS Part I		15.0 ± 7.1	10.9 ± 6.6	−4.0 ± 4.4	−25.7	<0.001
MDS-UPDRS Part II		13.1 ± 8.4	9.6 ± 6.9	−3.5 ± 4.7	−23.8	<0.001
MDS-UPDRS Part III		38.2 ± 14.4	27.8 ± 13.5	−10.4 ± 6.7	−28.5	<0.001
MDS-UPDRS Part IV		3.4 ± 3.2	2.2 ± 2.4	−1.2 ± 2.6	−30.1	0.004^c^
PIGD score		10.6 ± 6.2	7.5 ± 5.2	−3.1 ± 3.2	−27.9	<0.001
HYS	0	0 (0.0)	2 (4.8)			0.008^d^
	1	1 (2.4)	3 (7.1)			
	2	24 (57.1)	28 (66.7)			
	3	9 (21.4)	4 (9.5)			
	4	7 (16.7)	5 (11.9)			
	5	1 (2.4)	0 (0.0)			
Change in HYS after TMZ discontinuation	Improvement		16 (38.1)			
	No change		23 (54.8)			
	Worsening		3 (7.1)			
NMSS						
Cardiovascular problems		3.5 ± 3.7	2.4 ± 3.2	−1.1 ± 3.7	−28.6	0.053^c^
Sleep problems		14.3 ± 8.9	10.1 ± 7.3	−4.1 ± 7.3	−24.7	0.001
Mood problems		14.7 ± 17.0	11.4 ± 13.4	−3.3 ± 13.5	−19.7	0.119
Hallucinations		1.7 ± 3.9	1.8 ± 5.3	0.1 ± 5.3	5.1	0.931
Memory problems		6.7 ± 7.7	4.8 ± 5.9	−1.9 ± 6.9	−23.6	0.085
Gastrointestinal problems		5.5 ± 8.0	3.1 ± 5.2	−2.4 ± 6.7	−39.1	0.024
Urinary problems		12.2 ± 10.0	9.6 ± 8.7	−2.5 ± 8.5	−20.5	0.060
Sexual problems		0.9 ± 3.9	0.2 ± 0.5	−0.7 ± 3.9	−76.0	0.261^c^
Miscellaneous		3.4 ± 5.0	3.3 ± 4.6	−0.2 ± 4.0	−5.8	0.790^c^
Total		62.8 ± 49.9	46.6 ± 35.3	−16.2 ± 34.6	−25.6	0.004
MADRS		14.3 ± 8.8	11.4 ± 6.4	−3.0 ± 5.4	−20.1	0.001
PDSS-2		17.5 ± 10.9	15.1 ± 11.4	−2.4 ± 8.3	−13.8	0.068
ESS		6.4 ± 4.7	5.5 ± 4.7	−0.9 ± 5.0	−9.3	0.246
MoCA		22.2 ± 4.0	22.9 ± 4.0	0.5 ± 2.8	1.7	0.374
PDQ-39						
Mobility		30.2 ± 29.5	24.3 ± 23.6	−6.2 ± 18.9	−20.7	0.043
Activities of daily living		20.7 ± 23.7	15.2 ± 16.4	−5.7 ± 19.3	−27.2	0.067
Stigma		18.3 ± 27.3	13.1 ± 24.0	−5.6 ± 16.5	−26.2	0.034
Emotional well-being		25.5 ± 18.5	25.3 ± 18.0	−0.2 ± 13.0	−0.4	0.920
Social support		11.1 ± 14.0	6.9 ± 10.0	−4.5 ± 13.9	−37.2	0.046
Cognition		19.6 ± 16.3	19.1 ± 13.4	−0.8 ± 12.1	−3.7	0.692
Communication		14.7 ± 22.5	12.6 ± 21.3	−2.4 ± 17.9	−15.1	0.388
Bodily discomfort		36.3 ± 24.1	31.1 ± 23.0	−5.3 ± 22.1	−11.1	0.134
Summary index		21.9 ± 16.5	18.3 ± 13.9	−3.8 ± 10.8	−18.2	0.031

^a^Follow-up examinations were performed 95 ± 25 days after the baseline visit.

^b^To calculate relative change the following formula was used: (Score_Baseline_-Score_Follow-up_)/Score_Baseline_*100.

^c^The Wilcoxon signed rank test was used for the analysis of changes in these scores.

^d^The Chi-square test was utilized for the calculation of this value.

**Abbreviations: ESS** = Epworth Sleepiness Scale; **EQ** = EuroQoL instrument; **HYS** = Hoehn-Yahr Scale; **MADRS** = Montgomery-Asberg Depression Rating Scale; **MDS-UPDRS** = Movement Disorder Society-sponsored Unified Parkinson’s Disease Rating Scale; **MoCA** = Montreal Cognitive Assessment; **NMSS** = Non-Motor Symptoms Scale; **PDSS-2** = Parkinson’s Disease Sleep Scale 2^nd^ version; **PDQ-39** = 39-item Parkinson’s Disease Questionnaire; **PIGD** = postural instability and gait difficulty; **TMZ** = trimetazidine; **VAS** = visual analogue scale.

Most of the nonmotor symptoms also showed improvement, especially sleep problems and depression according to changes in the sleep domain of the NMSS (−4.1 points) and the total score of the MADRS (−3.0 points). In addition to symptomatic improvement, there was a significant rise in the HRQoL indicated by the change in the summary index of the PDQ-39 (−3.8 points).

Discontinuation of TMZ and modification of antianginal treatment did not lead to any cardiovascular events (e.g., acute ischaemic coronary syndrome, refractory angina pectoris) in the included patients during a 12-month FU.

## Discussion

Pharmacotherapy requires caution in patients with PD because a number of medications can worsen the symptoms of the disease. Harmful effects of inadequate use of antipsychotics in PD^1^, including increased mortality^29^, are well-documented; however, some other medication-related causes of worsening of PD have been less studied. Analyzing data of patients who were treated with TMZ despite a previously established clinical diagnosis of PD, the present study evaluated the effects of TMZ on the severity of symptoms of PD and the HRQoL by objectively determining the level of improvement after discontinuation of the drug.

According to changes in scores of the MDS-UPDRS, not only the severity of motor symptoms improved (Part III) but also the disability caused by both motor and nonmotor symptoms of PD moderated (Parts I and II) after the discontinuation of TMZ. These changes were not just statistically significant but also clinically relevant considering the minimal clinically important difference (MCID) threshold values for each part of the scale (2.64, 3.05, 3.25 and 0.9 points for improvement on Parts I, II, III and IV, respectively)^30–32^. Benefits of discontinuation of TMZ also manifested in a remarkable improvement of axial symptoms, which may indicate that TMZ worsens predominantly PIGD symptoms in patients with PD similarly to cases with reversible parkinsonism induced by the drug^5^. Axial symptoms are among the main sources of disability in PD and they can increase the risk of falls which can lead to additional problems including fractures and other injuries^33^, anxiety, depression, social isolation, and reduction of mobility^34^. Therefore, one of the main benefits of withdrawing TMZ in PD can be the improvement of the aforementioned problems.

The total score of the NMSS also detected a significant improvement in nonmotor symptoms. Of them, depression improved in a clinically relevant manner based on analysis of changes in total scores of the MADRS and considering the previously described MCID threshold value for this scale (from 1.6 to 1.9 points)^35^. Consequently, the use of TMZ in patients with PD and the negative impact of the drug on the severity of Parkinsonian symptoms seem to be clinically meaningful problems.

Current clinical research places a great emphasis on the exploration of the effects of disorders and therapeutic interventions on the HRQoL. Therefore, to measure the impact of TMZ on the HRQoL of patients with PD, changes in scores of the PDQ-39 due to the withdrawal of the drug were analyzed. Change in the summary index of the PDQ-39 indicated a statistically significant improvement in the global HRQoL. In addition, a substantial gain could be detected in the mobility domain of the PDQ-39. This may indicate that TMZ worsens predominantly bradykinesia, rigidity, and gait in patients with PD which is similar to the findings described in subjects with reversible TMZ-induced parkinsonism^5^. However, the change found by the present study did not exceed the MCID threshold for improvement established for the summary index of the PDQ-39 (4.72 points)^28^. A possible underlying cause for this ambiguous finding might be the relatively low number of included patients. Consequently, TMZ seems to have a negative impact on the HRQoL of patients with PD; however, further studies are warranted to clarify the clinical relevance of this phenomenon.

At present, a 24.5% improvement in the motor part of the MDS-UPDRS is considered to be a clinically relevant response to levodopa during an acute levodopa challenge^36^. Improvement in the severity of Parkinsonian motor symptoms achieved in the present study with the discontinuation of TMZ alone (28.5%) exceeded this threshold. Bilateral subthalamic deep brain stimulation, an established and highly effective treatment option for advanced PD, has been reported to improve the summary index of the PDQ-39 by 28% during the first 6 months of stimulation^37^. Compared to deep brain stimulation, withdrawal of TMZ led to a lower level of, however, a not negligible improvement (18.2%) in the global HRQoL in the present study. These findings also indicate the serious impact of TMZ on the severity of PD and the HRQoL and support the clinical importance of avoiding the use of TMZ in PD or the withdrawal of the drug in PD patients who are treated with it.

Some recent data and the present study show that the use of TMZ in patients with movement disorders is still an unsolved problem. According to the results of a recent population-based study, 2.5% of TMZ users are still patients with preexisting movement disorders^13^. The present study analyzed data of 42 patients with PD from the prospective database of the Movement Disorders Unit at the University of Pécs including data of 1800+ patients. Accordingly, approximately 2.3% of patients treated in our center were TMZ users prior to the study. However, such data may vary between centers and countries; therefore, studies involving analysis of data of a larger number of movent disorders centers and National Health Insurance databases of different countries are warranted for having a more reliable picture of the current clinical practice with TMZ.

Our results should be interpreted with caution since the study may have some limitations. First, we included patients representing a wide range of disease severity, however, a relatively low number of patients with moderate and severe PD was evaluated. Additionally, only patients treated with oral antiparkinsonian medications were enrolled, therefore, some interactions between TMZ and PD treated with device-aided therapy might have remained unexplored. Furthermore, the results of this single-center study might have been influenced by factors related specifically to the institution and/or the country.

To conclude, our results provide clinical rationale for avoiding the use of TMZ in PD. TMZ seems to worsen the severity of Parkinsonian symptoms in a clinically meaningful manner and have a negative impact on the HRQoL. Therefore, discontinuation of the drug in patients with PD seems to be a clinically adequate therapeutic intervention; however, prevention should have priority. Although TMZ is contraindicated for the treatment of patients with PD, in line with some recent data, the present study suggests that the number of PD subjects with TMZ use is still not negligible. However, larger scale studies should also evaluate the prevalence of TMZ prescriptions in patients with movement disorders, which could represent a more reliable picture of where we stand with the resolution of this still existing and clinically relevant problem.

## Data availability

Because the Ethical Approval of the present study does not authorize the authors to publish the data, they are not made available.

## References

[1] Ferreira JJ (2013). Summary of the recommendations of the EFNS/MDS-ES review on therapeutic management of Parkinson’s disease. Eur. J. Neurol..

[2] Shin HW, Chung SJ (2012). Drug-induced parkinsonism. J. Clin. Neurol..

[3] Montalescot G (2013). 2013 ESC guidelines on the management of stable coronary artery disease: the Task Force on the management of stable coronary artery disease of the European Society of Cardiology. Eur. Heart J..

[4] Ponikowski P (2016). ESC Guidelines for the diagnosis and treatment of acute and chronic heart failure: The Task Force for the diagnosis and treatment of acute and chronic heart failure of the European Society of Cardiology (ESC). Developed with the special contribution of the Heart Failure Association (HFA) of the ESC. Eur. J. Heart Fail..

[5] Pinter D (2019). Trimetazidine and parkinsonism: A prospective study. Parkinsonism Relat. Disord..

[6] Barre J (2003). Pharmacokinetic profile of a modified release formulation of trimetazidine (TMZ MR 35 mg) in the elderly and patients with renal failure. Biopharm. Drug. Dispos..

[7] Marti Masso JF, Marti I, Carrera N, Poza JJ, Lopez de Munain A (2005). Trimetazidine induces parkinsonism, gait disorders and tremor. Therapie.

[8] Marti Masso JF (2004). [Trimetazidine-induced parkinsonism]. Neurologia..

[9] Sommet A, Azais-Vuillemin C, Bagheri H, Rascol O, Montastruc JL (2005). Trimetazidine: a new cause for drug-induced parkinsonism?. Mov. Disord..

[10] Masmoudi K, Gras-Champel V, Douadi Y, Masson H, Andrejak M (2005). [Trimetazidine-a new aetiology for extrapyramidal disorders: A case of parkinsonism and akathisia]. Therapie.

[11] Masmoudi K, Masson H, Gras V, Andrejak M (2012). Extrapyramidal adverse drug reactions associated with trimetazidine: a series of 21 cases. Fundam. Clin. Pharmacol..

[12] Bondon-Guitton E (2011). Drug-induced parkinsonism: a review of 17 years’ experience in a regional pharmacovigilance center in France. Mov. Disord..

[13] Kwon J, Yu YM, Kim S, Jeong KH, Lee E (2019). Association between Trimetazidine and Parkinsonism: A Population-Based Study. Neuroepidemiol.

[14] Stephen PJ, Williamson J (1984). Drug-induced parkinsonism in the elderly. Lancet.

[15] Erbas O, Akseki HS, Elikucuk B, Taskiran D (2013). Antipsychotic-like effect of trimetazidine in a rodent model. Sci. World J..

[16] European Medicines Agency recommends restricting use of trimetazidine-containing medicines. Press release. European Medicines Agency (2012).

[17] Daniel SE, Lees AJ (1993). Parkinson’s Disease Society Brain Bank, London: overview and research. J. Neural Transm. Suppl..

[18] Litvan I (2003). Movement Disorders Society Scientific Issues Committee report: SIC Task Force appraisal of clinical diagnostic criteria for Parkinsonian disorders. Mov. Disord..

[19] Horvath K (2014). [Validation of the Hungarian Mds-Updrs: Why Do We Need a New Parkinson Scale?]. Ideggyogy. Sz..

[20] Goetz CG (2008). Movement Disorder Society-sponsored revision of the Unified Parkinson’s Disease Rating Scale (MDS-UPDRS): scale presentation and clinimetric testing results. Mov. Disord..

[21] Stebbins GT (2013). How to identify tremor dominant and postural instability/gait difficulty groups with the movement disorder society unified Parkinson’s disease rating scale: comparison with the unified Parkinson’s disease rating scale. Mov. Disord..

[22] Goetz CG (2004). Movement Disorder Society Task Force report on the Hoehn and Yahr staging scale: status and recommendations. Mov. Disord..

[23] Kovacs M (2016). Impact of Sex on the Nonmotor Symptoms and the Health-Related Quality of Life in Parkinson’s Disease. Parkinsons Dis..

[24] Kaszas B (2012). Sensitivity and specificity of Addenbrooke’s Cognitive Examination, Mattis Dementia Rating Scale, Frontal Assessment Battery and Mini Mental State Examination for diagnosing dementia in Parkinson’s disease. Parkinsonism Relat. Disord..

[25] Horvath K (2014). Test-retest validity of Parkinson’s disease sleep scale 2nd version (PDSS-2). J. Parkinsons Dis..

[26] Hagell P, Broman JE (2007). Measurement properties and hierarchical item structure of the Epworth Sleepiness Scale in Parkinson’s disease. J. Sleep. Res..

[27] Lucza T (2015). Screening Mild and Major Neurocognitive Disorders in Parkinson’s Disease. Behav. Neurol..

[28] Horvath K (2017). Changes in Quality of Life in Parkinson’s Disease: How Large Must They Be to Be Relevant?. Neuroepidemiol.

[29] Weintraub D (2016). Association of Antipsychotic Use With Mortality Risk in Patients With Parkinson Disease. JAMA Neurol..

[30] Horvath K (2017). Minimal clinically important differences for the experiences of daily living parts of movement disorder society-sponsored unified Parkinson’s disease rating scale. Mov. Disord..

[31] Horvath K (2015). Minimal clinically important difference on the Motor Examination part of MDS-UPDRS. Parkinsonism Relat. Disord..

[32] Makkos A, Kovacs M, Pinter D, Janszky J, Kovacs N (2019). Minimal clinically important difference for the historic parts of the Unified Dyskinesia Rating Scale. Parkinsonism Relat. Disord..

[33] Wielinski CL, Erickson-Davis C, Wichmann R, Walde-Douglas M, Parashos SA (2005). Falls and injuries resulting from falls among patients with Parkinson’s disease and other parkinsonian syndromes. Mov. Disord..

[34] Bloem BR, Hausdorff JM, Visser JE, Giladi N (2004). Falls and freezing of gait in Parkinson’s disease: a review of two interconnected, episodic phenomena. Mov. Disord..

[35] Duru G, Fantino B (2008). The clinical relevance of changes in the Montgomery-Asberg Depression Rating Scale using the minimum clinically important difference approach. Curr. Med. Res. Opin..

[36] Pinter D, Martinez-Martin P, Janszky J, Kovacs N (2019). The Parkinson’s Disease Composite Scale Is Adequately Responsive to Acute Levodopa Challenge. Parkinsons Dis..

[37] Siderowf A (2006). Long-term effects of bilateral subthalamic nucleus stimulation on health-related quality of life in advanced Parkinson’s disease. Mov. Disord..

